# Clinical impact of post-progression survival on overall survival in patients with limited-stage disease small cell lung cancer after first-line chemoradiotherapy

**DOI:** 10.1515/raon-2015-0037

**Published:** 2015-11-27

**Authors:** Norimitsu Kasahara, Hisao Imai, Kyoichi Kaira, Keita Mori, Kazushige Wakuda, Akira Ono, Tetsuhiko Taira, Hirotsugu Kenmotsu, Hideyuki Harada, Tateaki Naito, Haruyasu Murakami, Masahiro Endo, Takashi Nakajima, Masanobu Yamada, Toshiaki Takahashi

**Affiliations:** 1 Division of Thoracic Oncology, Shizuoka Cancer Center, Japan; 2 Clinical Trial Coordination Office, Shizuoka Cancer Center, Japan; 3 Division of Radiation Oncology, Shizuoka Cancer Center, Japan; 4 Division of Diagnostic Radiology, Shizuoka Cancer Center, Japan; 5 Division of Diagnostic Pathology, Shizuoka Cancer Center, Japan; 6 Department of Medicine and Molecular Science, Gunma University Graduate School of Medicine, Japan; 7 Department of Oncology Clinical Development, Gunma University Graduate School of Medicine, Japan

**Keywords:** chemoradiotherapy, limited-stage disease small cell lung cancer, overall survival, post-progression survival, progression-free survival

## Abstract

**Background:**

The effects of first-line chemoradiotherapy on overall survival (OS) may be confounded by subsequent lines of therapy in patients with limited-stage disease small cell lung cancer (LD-SCLC). Therefore, we aimed to determine the relationships between progression-free survival (PFS), post-progression survival (PPS) and OS after first-line chemoradiotherapy in LD-SCLC patients.

**Patients and methods.:**

We retrospectively analyzed 71 LD-SCLC patients with performance status (PS) 0–2 who received first-line chemoradiotherapy and had disease recurrence between September 2002 and March 2013 at Shizuoka Cancer Center (Shizuoka, Japan). We determined the correlation between PFS and OS and between PPS and OS at the individual level. In addition, we performed univariate and multivariate analyses to identify significant prognostic factors of PPS.

**Results:**

OS is more strongly correlated with PPS (Spearman’s *r* = 0.86, *R*^2^ = 0.72, *p* < 0.05) than PFS (Spearman’s *r* = 0.46, *R*^2^ = 0.38, *p* < 0.05). In addition, the response to second-line treatments, the presence of distant metastases at recurrence and the number of additional regimens after first-line chemoradiotherapy were significant independent prognostic factors for PPS.

**Conclusions:**

PPS has more impact on OS than PFS in recurrent LD-SCLC patients with good PS at beginning of the treatment. Moreover, treatments administered after first-line chemoradiotherapy may affect their OS. However, larger multicenter studies are needed to validate these findings.

## Introduction

Lung cancer is the leading cause of cancer-related mortality worldwide.^1^ In the United States, 14% of people who were diagnosed with lung cancer had small cell lung cancer (SCLC).^2^ Approximately 30% of SCLC patients have limited-stage disease small cell lung cancer (LD-SCLC), which is characterized by locoregional tumors in the hemithorax, mediastinum, or supraclavicular lymph nodes, while the rest have extensive-stage disease.^3^ Current therapeutic options for LD-SCLC include combination chemotherapy with etoposide and cisplatin, chest radiotherapy, and prophylactic cranial irradiation (PCI).^4^,^5^ However, due to the limited efficacy of these therapeutic strategies and the aggressive nature of SCLC tumors, the prognosis for SCLC patients is poor; the median survival time for LDSCLC patients is less than two years.^6^–^8^

PFS and OS are two common endpoints in cancer trials. OS is usually preferred, because it is reliable, precise, meaningful and easily documented by the date of death.^9^ However, the effect of first-line treatments on OS might be confounded by subsequent lines of therapy. In contrast, PFS is quicker to measure, can be measured more conveniently, and therefore, may be easier to assess than OS.^10^ If there is a strong correlation between PFS and OS, then PFS may be a surrogate endpoint for OS. In non-small cell lung cancer (NSCLC), increases in PFS do not necessarily increase OS, but post-progression survival (PPS) is strongly associated with OS after first-line treatment.^11^–^13^ We have also demonstrated a strong correlation between PPS and OS after first-line chemotherapy in patients with extensive-stage disease SCLC.^14^ In LD-SCLC, though, the relationship between PPS and OS is unknown.

Therefore, we analyzed the correlation between PFS and OS and between PPS and OS after first-line chemoradiotherapy in LD-SCLC patients to determine whether PFS or PPS has more influence on OS. We also investigated the prognostic value of baseline and tumor characteristics for PPS.

## Patients and methods

### Patients

We retrospectively enrolled 71 consecutive patients with recurrent LD-SCLC after receiving first-line chemoradiotherapy at Shizuoka Cancer Center (Shizuoka, Japan) between September 2002 and March 2013. The inclusion criteria were as follows: (1) histologically or cytologically confirmed SCLC; (2) 20 years of age or older at the time of chemoradiotherapy; (3) Eastern Cooperative Oncology Group performance status (PS) of 0–2 at the beginning of the first-line treatment; (4) first-line treatment with ≥ 40 Gy curative thoracic radiotherapy and platinum doublet chemotherapy, either concurrently or sequentially; and (5) disease recurrence after first-line treatment. The study protocol was approved by the Institutional Review Board of Shizuoka Cancer Center and was conducted in accordance with the Helsinki Declaration of 1964 (revised 2008). Due to the retrospective nature of this study, the need for informed consent was waived.

### Treatment

LD-SCLC patients were treated with a combination of chemotherapy and radiotherapy. Several different first-line chemotherapeutic regimens were used; etoposide (80 or 100 mg/m^2^) was administered on days 1–3 in combination with cisplatin (80 mg/m^2^) on day 1, cisplatin (25 mg/m^2^) on days 1–3, or carboplatin (area under the curve = 5) on day 1. These drugs were injected intravenously every 3–4 weeks for maximum 4 courses. Second and third-line treatments included amrubicin, irinotecan, topotecan, gemcitabine, and paclitaxel.

The fractionation schedule for thoracic radio-therapy in LD-SCLC patients was determined by using information from chest computed tomography (CT) to calculate the pretreatment tumor volume. Typically, the total planned dose was 50 Gy when it was fractionated once daily or 45 Gy when it was fractionated twice daily, based on individual physician decision. Furthermore, the maximum spinal cord dose was limited to 45 Gy when the radiation dose was fractionated once daily or to 36 Gy when it was fractionated twice daily. In addition, no more than 35% of the normal lung volume received more than 20 Gy.

Thoracic radiotherapy was started either during the first cycle of chemotherapy or after four cycles of chemotherapy. It was suspended if a patient experienced grade 4 thrombocytopenia, neutropenia, radiation pneumonitis, fever caused by infection, a decrease of more than 10 mmHg in arterial oxygen pressure, or difficulty swallowing liquids. After thoracic radiotherapy, PCI (25 Gy in 10 fractions) was administered to patients with a complete or near-complete response, as shown by a scar-like shadow on a chest CT, if the treating physician recommended it.

### Assessment of treatment efficacy

Tumor responses reflect the best overall response and maximum shrinkage. Radiographic tumor responses were evaluated using chest computed tomography at every two courses of chemotherapy according to the Response Evaluation Criteria In Solid Tumors 1.0 as follows: complete response (CR), disappearance of all target lesions; partial response (PR), ≥ 30% decrease in the total diameter of all target lesions relative to the total baseline diameter; progressive disease (PD), ≥ 20% increase in the total diameter of all target lesions relative to the smallest total diameter observed during the study; and stable disease (SD), insufficient change in the total diameter of all target lesions to qualify as PR or PD.^15^

PFS was defined as the time from the beginning of first-line treatment until documented PD or death. In addition, OS was reported as the time from the beginning of first-line treatment until death or censored at the time of the last assessment of disease status. Similarly, PPS was documented as the time from tumor progression after first-line treatment until death or censored at the time of the last assessment of disease status.

### Treatment-free interval

In this study, we defined treatment-free interval (TFI) as the period from the date of completion of first-line treatment to first relapse. When sequential radiotherapy or PCI were performed as first-line treatment, the date of completion of first-line treatment was defined as the last day of these treatments.

Since TFI is known as a predictive factor of second-line chemotherapy, we analyzed patients according to TFI.^16^, ^17^ In many trials, the relapsed SCLC patients with TFI more than 90 days were defined as sensitive relapses. This definition was also used in this study.

### Statistical analyses

We used Spearman’s rank correlation and linear regression analyses to determine whether PFS or PPS correlated with OS in LD-SCLC patients. We also applied the Cox proportional hazards model with a stepwise regression procedure to determine prognostic factors for PPS and estimate hazard ratios and 95% confidence intervals. The effects of different prognostic factors on PPS were compared using the log-rank test. *P*-values less than 0.05 were considered to be statistically significant for both one-tailed and two-tailed tests. All statistical analyses were performed using JMP (version 11.0; SAS Institute, Cary, NC, USA).

## Results

### Patient characteristics and treatment efficacy

**FIGURE 1.**
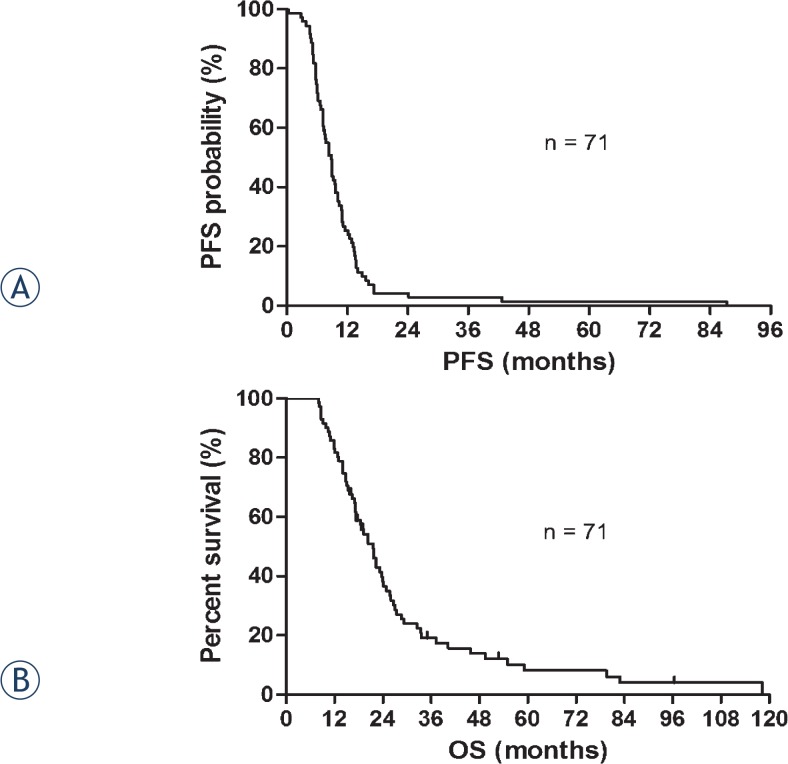
Kaplan-Meier survival plots of **(A)** progression-free survival (PFS) and **(B)** overall survival (OS) in 71 limited-stage disease small cell lung cancer (LD-SCLC) patients in this study. Median PFS: 8.8 months, median OS: 21.6 months, median follow-up period: 19.1 months.

Between September 2002 and March 2013, 116 patients with LD-SCLC were treated with chemoradiotherapy, and 71 patients who recurred after first-line treatment were enrolled in this study. Patient characteristics are summarized in Table 1. The majority of patients (80.3%) received concurrent chemotherapy and radiotherapy. Cisplatin plus etoposide combination chemotherapy was the most common first-line treatment. Subsequently, 21/71 (29.6%) patients received a median of one additional regimen (range: 0–6). Twenty-one patients temporarily interrupted RT, but all of them completed previously planned radiation doses. During a median follow-up period of 19.1 months (range: 8.0–118.3 months), 63/71 (88.7%) patients died. Nine patients experienced a CR, 56 patients had a PR, three patients showed SD, and three patients exhibited PD. The overall response rate was 91.5% and the disease control rate was 95.7%. The median PFS and OS were 8.8 months and 21.6 months, respectively (Figures 1A, 1B). The mean OS of other 45 patients who didn’t experience recurrence after first-line treatment was 46.5 months (median not reached).

### Prognostic factors for post-progression survival

**FIGURE 2.**
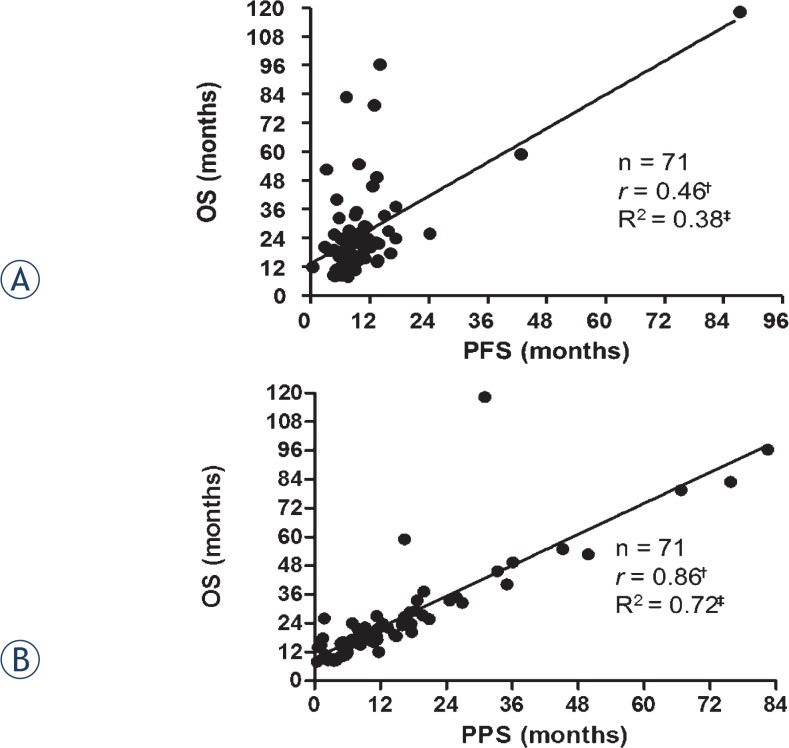
Correlations between overall survival (OS) and **(A)** progression-free survival (PFS) and **(B)** post-progression survival (PPS) in 71 limited-stage disease small cell lung cancer (LD-SCLC) patients. ^†^Spearman’s rank correlation coefficient. ^‡^Linear regression correlation coefficient.

Since OS was more strongly correlated with PPS (Spearman’s *r* = 0.86, *R*^2^ = 0.72, *p* < 0.05; Figure 2B) than PFS (Spearman’s *r* = 0.46, *R*^2^ = 0.38, *p* < 0.05; Figure 2A), we assessed the significance of potential prognostic factors for PPS. Univariate analysis showed that six factors, namely, age at the beginning of first-line treatment, relative timing of chemotherapy and radiotherapy (sequential vs. concurrent), response to second-line treatment (non PD vs. PD), the presence of distant metastases at recurrence (yes vs. no), administration of platinum-based chemotherapeutic agents after first-line treatment (yes *vs*. no), and the number of regimens after first-line treatment, were significantly associated with PPS (*p* < 0.05; Table 2). However, multivariate analysis revealed that only the response to second-line treatment (non PD vs. PD), the presence of distant metastases at recurrence (yes *vs*. no) and the number of additional regimens after first-line treatment are significant independent prognostic factors for PPS (Table 3).

**FIGURE 3.**
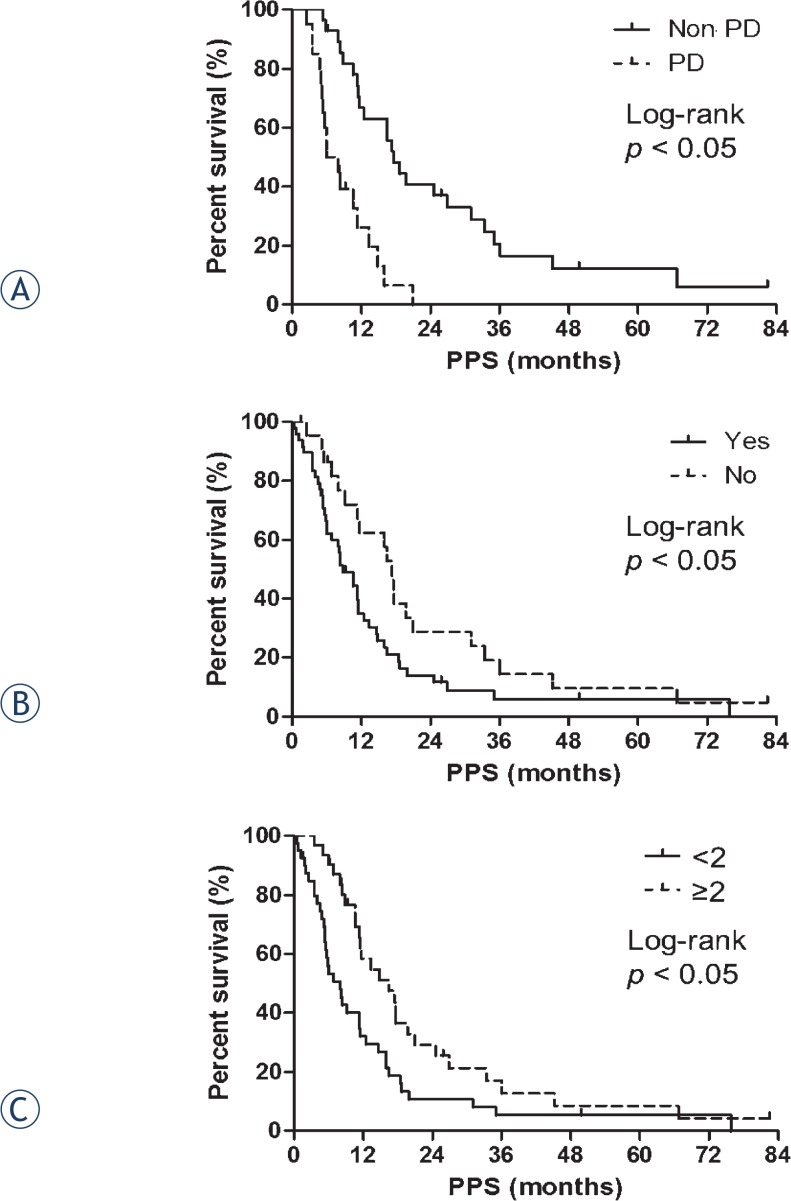
Three significant independent prognostic factors of post-progression survival (PPS) (Table 3) result in significantly different PPS distributions in 71 limited-stage disease small cell lung cancer (LD-SCLC) patients (log rank test, *p* < 0.05). **(A)** Response to second-line treatment (progressive disease [PD] vs. non progressive disease [non PD]). Median PPS for non PD: 17.5 months vs. PD: 6.9 months. **(B)** Presence of distant metastases at recurrence (Yes vs. No). Median PPS for Yes: 8.7 months vs. No: 17.3 months. **(C)** Number of regimens after first-line treatment. Median PPS for ≥ 2 additional regimens: 16.0 months vs. < 2 regimens: 6.8 months.

We used these three prognostic factors to construct Kaplan-Meier plots of PPS (Figures 3A, 3B and 3C), which showed that the survival distributions for response to second-line treatment (non PD *vs*. PD), the presence of distant metastases at recurrence (yes *vs*. no) and the number of additional regimens after first-line treatment (< 2 *vs*. ≥ 2) are significantly different (log-rank tests, *p* < 0.05). Specifically, the median PPS in patients without PD after second-line treatment (17.5 months) was significantly greater than that for patients with PD (6.9 months; *p* < 0.05). Furthermore, the median PPS in patients without distant metastases (17.3 months) was significantly greater than that for patients with distant metastases (8.7 months; *p* < 0.05). In addition, the median PPS of patients who received two or more regimens after first-line treatment (16.0 months) was significantly greater than that for patients who received less than two additional regimens (6.8 months; *p* < 0.05).

## Discussion

In this study, we examined the relationships between OS and PFS or PPS, for recurrent LD-SCLC patients after first-line chemoradiotherapy and found that OS correlates more strongly with PPS than PFS. In addition, we determined that the response to second-line treatment, the presence of distant metastases at recurrence and the number of additional regimens after first-line treatment are significant independent prognostic factors for PPS. To our knowledge, this is the first report of individual-level factors that affect PPS for LD-SCLC patients after first-line chemoradiotherapy.

Several previous meta-analyses have assessed the value of surrogate endpoints, such as time to progression for survival in cancer studies.^18^,^19^ In extensive-stage disease SCLC, tumor response and PFS have been proposed as potential surrogate endpoints for OS, but their appropriateness is controversial in LD-SCLC.^20^ Computer simulations have shown that significance of OS may be diluted if PPS is long.^9^ Other studies have also demonstrated that PPS is strongly correlated with OS for advanced NSCLC after both first-line chemotherapy and subsequent lines of therapy.^12^,^13^, ^21^ Similarly, we have previously reported that PPS is a potential surrogate marker for advanced NSCLC and extensive-stage disease SCLC.^14^, ^22^

Our finding that OS is more strongly correlated with PPS than PFS implies that subsequent treatments have more effects on OS than the first line treatment. Therefore, LD-SCLC clinical trials should account for factors that may affect PPS to avoid confounding OS. Actually, this recommendation may apply to SCLC in general, because the two of three significant independent prognostic factors associated with PPS for LD-SCLC patients that we identified in this study, namely, response to second-line treatment and the number of additional regimens after first-line chemotherapy, are also associated with PPS in extensive-stage SCLC patients.^14^

These prognostic factors for PPS also suggest that disease stabilization after disease progression following first-line chemoradiotherapy may allow LD-SCLC patients to receive additional lines of treatment, which could prolong PPS, and consequently, OS. Although a number of treatment choices in SCLC are less than that of NSCLC, the large number of treatment regimens that were used after first-line chemoradiotherapy in this study is mainly due to the increasing number of chemo-therapeutic options, such as amrubicin, irinotecan, and topotecan, for subsequent-line chemotherapy for LD-SCLC. However, treatments with platinum-based chemotherapeutic agents and amrubicin after first-line treatment were not significant prognostic factors for PPS, which suggest that these drugs do not affect PPS or OS. Likewise, treatment with sequential or concurrent chemoradiotherapy was not a significant prognostic factor for PPS; however, relative few patients in this study were treated sequentially, so there may have been insufficient statistical power to detect a significant difference.

This study has three major limitations. First, the sample size was relatively small. This limitation is difficult to overcome, particularly in studies that analyze patients with similar backgrounds, because there are relatively few LD-SCLC patients at any given institution. Nevertheless, our institution treats a fair number of these cases and uses unified treatment regimens. Second, the single-center design of our study may limit the generality of our conclusions, so multicenter trials are needed to validate our results in larger patient populations and other clinical settings. Third, since different physicians documented patient responses, the timing of evaluation of PFS and tumor response rates may have been less accurate than if only a single physician had documented all responses. However, this is one of the major limitations of retrospective study, and it is unavoidable. Prospective trials are needed to investigate the validity.

In conclusion, PPS has more impact on OS than PFS in recurrent LD-SCLC patients after first-line chemoradiotherapy. In addition, the response to second-line treatment, the presence of distant metastases at recurrence and the number of additional regimens after first-line treatment are significant independent prognostic factors for PPS. These results suggest that treatments administered after first-line chemoradiotherapy affect OS in LD-SCLC patients. However, larger multicenter studies are needed to validate these conclusions in other patient populations and clinical settings.

## Figures and Tables

**TABLE 1.** Patient characteristics

**Characteristic**	**Patients (*n*)**
Gender	
Male	57
Female	14
Age (years)	
Median	69
Range	45–92
Performance status	
0	32
1	37
2	2
Clinical stage	
II	8
III	63
Tumor histology	
Small cell carcinoma	68
Combined small cell carcinoma	3
Smoking history	
Current or former smoker	70
Never smoked	1
Number of first-line chemotherapy courses	
1	2
2	1
3	4
4	63
5	1
Number of regimens after first-line treatment	
0	18
1	21
2	16
3	8
4	4
5	2
6	2
Radiation dose (Gy)	
Median	45
Range	40–60
Chemoradiotherapy	
Concurrent	56
Sequential	15
First-line chemotherapy regimens	
Cisplatin + etoposide	49
Carboplatin + etoposide	18
Cisplatin + etoposide → Cisplatin + irinotecan	3
Cisplatin + etoposide → Cisplatin + Vincristine + Doxorubicin + etoposide	
Subsequent lines of chemotherapy, total (second-line/third-line or more)	
Platinum combination	25 (15/10)
Amrubicin	36 (22/14)
Irinotecan	25 (9/16)
Topotecan	13 (7/6)
Gemcitabine	7 (0/7)
Paclitaxel	6 (0/6)
Investigational drug	2 (0/2)
Distant metastases at recurrence	
Yes	48
No	23
Prophylactic cranial irradiation	
Yes	27
No	44

**TABLE 2.** Univariate analysis of factors associated with post-progression survival in limited-stage small cell lung cancer patients

**Factors**	**Post-progression survival**		

	**Hazard ratio**	**95% CI**	***p*-value**
Gender	1.42	0.78–2.81	0.25
Age (years) at the beginning of first-line treatment	1.03	1.00–1.06	**0.03**
Age (years) at the beginning of second-line treatment	1.02	0.99–1.06	0.10
PS at the beginning of first-line treatment	0.90	0.55–1.48	0.69
PS at the end of first-line treatment	0.77	0.47–1.25	0.29
PS at the beginning of second-line treatment	1.31	0.83–2.03	0.23
Tumor histology (small cell carcinoma/combined small cell carcinoma)	1.55	0.63–5.16	0.36
Clinical stage at the beginning of first-line treatment (II/III)	0.55	0.22–1.15	0.12
Chemoradiotherapy (sequential/concurrent)	2.21	1.14–3.99	**0.01**
Number of courses of first-line chemotherapy	1.08	0.75–1.77	0.69
Best response at first-line treatment			
PR/ nonPR	0.98	0.45–2.57	0.97
NonPD /PD	1.33	0.49–5.48	0.61
Best response at second-line treatment			
PR/ nonPR	0.63	0.31–1.21	0.17
NonPD/PD	0.23	0.11–0.45	**< 0.01**
Treatment-free interval Sensitive/refractory	0.87	0.49–1.64	0.65
Distant metastases at recurrence (yes/no)	1.77	1.05–3.10	**0.03**
Administration of platinum-based agents after first-line treatment (yes/no)	0.51	0.28–0.88	**0.01**
Administration of amrubicin after first-line treatment (yes/no)	0.71	0.39–1.28	0.25
Prophylactic cranial irradiation (yes/no)	0.75	0.44–1.25	0.28
Number of regimens after first-line treatment	0.84	0.71–0.98	**0.02**

CI = confidence interval; PD = progressive disease; PR = partial response; PS = performance status. Boldfaced *p*-values are statistically significant (*p* < 0.05).

**TABLE 3.** Multivariate analysis of factors associated with post-progression survival in limited-stage small cell lung cancer patients

**Factors**	**Post-progression survival**		

	**Hazard ratio**	**95% CI**	***p*-value**
Age (years) at the beginning of first-line treatment	0.98	0.94–1.02	0.47
Chemoradiotherapy (sequential/concurrent)	2.25	0.66–7.04	0.18
Best response at second-line treatment (NonPD/PD)	0.22	0.10–0.47	**< 0.01**
Distant metastases at recurrence (yes/no)	2.42	1.18–5.22	**0.01**
Administration of platinum-based agents after first-line treatment (yes/no)	0.92	0.41–1.98	0.83
Number of regimens after first-line treatment	0.75	0.56–0.98	**0.04**

CI = confidence interval; PD = progressive disease. Boldfaced *p*-values are statistically significant (*p* < 0.05).
